# Life in a time of COVID: retrospective examination of the association between physical activity and mental well-being in western Australians during and after lockdown

**DOI:** 10.1186/s12889-023-15440-1

**Published:** 2023-04-14

**Authors:** Ben Piggott, Paola Chivers, Kiira Karoliina Sarasjärvi, Ranila Bhoyroo, Michelle Lambert, Lynne Millar, Caroline Bulsara, Jim Codde

**Affiliations:** 1 School of Health Sciences, Faculty of Medicine, Midwifery & Health Sciences, The University of Notre Dame Australia, Nursing, Fremantle, Australia; 2 Institute for Health Research, The University of Notre Dame Australia, Fremantle, Australia; 3 School of Medical and Health Sciences, Edith Cowan University, Joondalup, Australia; 4 DATaR Consulting, Bridgetown, Australia; 5 Telethon Kids Institute, Nedlands, Australia; 6 Discipline of Psychology and Exercise Science, Murdoch University, Murdoch, Australia; 7 Division of Obstetrics and Gynaecology, Faculty of Health and Medical Sciences, University of Western Australia, Crawley, Australia; 8 Faculty of Medicine, Doctoral Programme in Population Health, University of Helsinki, Helsinki, Finland

**Keywords:** Physical activity, COVID-19, Pandemic, Health promotion, Western Australia, Lockdown

## Abstract

**Background:**

The aim of this study was to examine physical activity and sedentary behaviours during Western Australia’s COVID-19 lockdown and their association with mental well-being.

**Methods:**

Participants completed activity related questions approximately two months after a three-month lockdown (which formed part of a larger cross-sectional study from August to October 2020) as part of a 25-minute questionnaire adapted from the Western Australia Health and Well-being Surveillance system. Open-ended questions explored key issues relating to physical activity behaviours.

**Results:**

During the lockdown period, 463 participants (female, *n* = 347; 75.3%) reported lower number of active days (W = 4.47 p < .001), higher non-work-related screen hours per week (W = 11.8 p < .001), and higher levels of sitting time (χ^2^=28.4 p < .001). Post lockdown body mass index was higher (U = 3.0 p = .003), with obese individuals reporting the highest non-work-related screen hours per week (Wald χ^2^= 8.9 p = .012). Inverse associations were found for mental well-being where higher lockdown scores of Kessler-10 (p = .011), Dass-21 anxiety (p = .027) and Dass-21 depression (p = .011) were associated with lower physical activity levels. A key qualitative message from participants was wanting to know how to stay healthy during lockdown.

**Conclusions:**

Lockdown was associated with lower physical activity, higher non-work-related screen time and more sitting time compared to post lockdown which also reported higher body mass index. Lower levels of mental well-being were associated with lower physical activity levels during lockdown. Given the known positive affect of physical activity on mental well-being and obesity, and the detrimental associations shown in this study, a key public health message should be considered in an attempt to maintain healthy activity behaviours in future lockdowns and similar emergency situations to promote and maintain positive well-being. Furthermore, consideration should be given to the isolation of a community due to infectious disease outbreaks and to recognise the important role physical activity plays in maintaining weight and supporting good mental health.

**Supplementary Information:**

The online version contains supplementary material available at 10.1186/s12889-023-15440-1.

## Introduction

Since its emergence in 2019, the COVID-19 pandemic has had an enormous impact on everyday lives. Across the world, governments used a variety of strategies in an attempt to reduce the spread of the virus, including lockdowns and community restrictions. Whilst lockdowns and community restrictions may reduce infection rates, it also impacts the ability to participate in physical activity. It is well known that being physically active has a multitude of benefits including being associated with reduced chronic disease risk [1]. In recognition of the importance that physical activity plays in contributing to optimum health, the World Health Organisation (WHO) has produced recommendations in regards to amount of physical activity people should undertake. WHO recommend at least 150 min of moderate intensity physical activity throughout the week, or at least 75 min of vigorous intensity physical activity throughout the week and muscle-strengthening activities involving major muscle groups should be done on two or more days a week [2]. Similar guidelines are also produced by the Australian Government who recommend that adults aged 18–64 years, complete either 2.5 to 5 h of moderate intensity physical activity (such as a brisk walk, golf, mowing the lawn or swimming) or 1.25 to 2.5 h of vigorous intensity physical activity (such as jogging, aerobics, fast cycling, soccer or netball) or an equivalent combination of moderate and vigorous activities [3].

The rate of infection and resultant deaths, as well as the strategies to control the spread of the virus have varied across countries. Australia employed strategies such as widespread testing, contact tracing, border closures and lockdowns as the major tools to reduce community transmission and the COVID-19 waves have been small, compared to many other countries [4]. The impact of the lockdowns, which included stringent government mandated community level restrictions, on the community’s overall health is emerging. Previously reported cross-sectional research with this cohort described the impact of COVID-19 on a group of West Australians reporting that lockdown was associated with a significantly lower levels of physical activity, poorer mental well-being and sense of control over one’s life, and a higher level of loneliness [5]. Furthermore, it was also found that lockdown resulted in a significantly higher consumption of junk food, alcoholic drinks and soft drinks but no change in fruit and vegetable intake.

[5]. This paper extends these findings by exploring physical activity behaviours throughout the lockdown and their influence on mental well-being. New qualitative data is presented to help explain potential drivers identified. Importantly these findings are used to provide specific recommendations to inform future educational campaigns for physical activity during future lockdown scenarios.

The strictness of lockdown and community restrictions varied throughout the world; however a common element in many COVID-19 management strategies was the closure of non-essential business including gyms and the cessation of community sport which occurred in Australia [4] and these changes impacted people’s ability to be physically active in various ways. A systematic review from Stockwell et al. [6], reported an increase in physical inactivity during the COVID-19 lockdown period, with all 26 studies reporting changes in physical activity and increase in sedentary behaviour, respectively. Moreover, studies have shown association between physical activity/inactivity and mental health outcomes during the lockdown period [6–9]; yet more specific detail needs to be explored in regards to this association with different populations.

Recently studies have reported a range of the impacts of COVID-19 and mandated lockdowns. Even though some studies have reported an overall increase in inactive behaviour during COVID-19, a relatively large portion of people have also reported to have no change in their behaviour [6], emphasizing the importance of individual differences. Similarly, although we previously reported overall significantly lower levels of physical activity during the lockdown period, this comprised three distinct groups; a third lower, one-fifth higher, and half not changing their levels of physical activity during and post-lockdown [5].

In general, physical inactivity (e.g., low physical activity, high sedentary behaviour) have severe health outcomes and are associated with potentially increased risk of premature mortality and several chronic medical conditions, such as dementia, as well as mental health disorders, and cardio-metabolic and -vascular disease [8, 10–12]. Health professionals have pinpointed the reduction on physical activity and increased mental health issues resulting from COVID-19 related restrictions, as major concerns [13]. Importantly, Stockwell et al. [6] acknowledged the importance of understanding the changes in activity behaviours during lockdowns in order to facilitate the development of public health interventions for ongoing future pandemic scenarios. This study aims to explore behaviours that were protective for maintaining physical activity and minimising adverse impacts to mental well-being and how they might inform future health promotion campaigns.

The setting context for this study was a key government strategy to minimise COVID-19 transmission; community lockdown, characterised by an initial rapid closing of national and Western Australian state borders to travellers in March 2020. Direct potential impacts on physical activity behaviours included the closure of social venues (sporting clubs and venues, gyms and fitness centres, cinemas), with limitations of one hour outside exercise per day mandated. Additionally, the work from home, and travel restrictions for essential services only reduced incidental activity levels through active transport (e.g., walking to public transport, taking the stairs at workplaces). From mid-May through to late June 2020, these restrictions began to ease and by March 2021, the Western Australian community was operating relatively normally, although some short duration lockdowns (days) continued to be enacted [5, 14]. Throughout this period COVID-19 infections rates in Western Australia remained low compared to national and international rates [15].

Therefore, the aim of this study was to examine in depth, physical activity, screen time, and sedentary behaviours of the general population in Western Australians during the first significant state COVID-19 lockdown period and their potential associations with mental health factors. Our overarching research question was how had mandated lockdown isolation impacted on people, their lifestyle and well-being and are there potential associations between physical inactivity and mental well-being among Western Australians during and after COVID-19-Lockdown.

## Methods

### Study design

This mixed method study reports on the physical activity components of the overarching health and well-being survey previously reported [5] and hence employs the same methodology. Briefly, residents within Western Australian aged 18 years or older were invited through a range of community and social media mechanisms to complete a cross-sectional study using a web-based survey across several health and well-being factors. Survey responses were collected over an 8-week period (between 25th August – 21st October 2020) approximately two months after an enforced community lockdown of three months duration (23rd March – 27th June 2020) [5]. The study was granted ethical approval by the institutional Health and Research Ethics Committee (2020-133 F).

*A priori* sample size calculation was conducted for the overarching health and well-being survey. The minimum sample of 174 participants was based on between group differences (e.g., five age groups) using a medium effect size of 0.31, *α* = 0.05, and a power = 0.80. This *a priori* sample size was determined to also enable lockdown and post-lockdown response comparisons (G*Power version 3.1.9.2, Kiel, Germany; 2014). This study reports on a subset of participants (n = 463) who reported at least one physical activity measure (activity levels, active days) drawn from the overarching Health and Well-being study (n = 547) [5].

### Questionnaire design

The Health and Well-being questionnaire was completed by participants using an online platform (Qualtrics, Provo, UT) taking on average 25 min to complete with full study design and survey previously reported [5]. The survey comprised several sections of questions, including open ended questions. Basic socio-demographic information was collected on sex, age, postcode, locality, level of education, employment status and household income.

Lifestyle behaviours (physical activity, sedentary behaviours) questions were adopted from the Western Australia Health and Well-being Surveillance system (WA-HWSS) survey [16]. This study focussed on the physical activity and sedentary behaviours components of the survey and their associations with mental well-being. Physical activity was assessed as current/over the past week (time of questionnaire completion) and in retrospect (thinking back to during/typical week of COVID-19 lockdown). Participants were asked to rate their level of physical activity (very active, active, moderately active, not very active and not active at all); and how many days they were physically active for at least 30 min per day (1–7 days). Physical activity levels were also described between lockdown to post-lockdown as being the same, higher or lower physical activity levels. In a series of open-ended question, participants were asked to think back to the lockdown period and describe (a) what was the biggest difference to their physical activity, (b) if they did any physical activity related purchases (e.g. home treadmill), and (c) if they had any changes to their activity levels, habits or choices.

Sedentary behaviour was assessed as current and thinking back to during COVID-19 lockdown. Participants were asked to rate how they spent most of their day (mostly sitting, mostly standing, mostly walking, or mostly doing heavy labour or physically demanding work); and time (hours and minutes) spent per week (excluding work time) watching TV or DVDs, or using the computer, iPad or tablet device (for the internet, to play games etc.). In a series of open-ended question participants were asked to describe changes in their screen and leisure time.

The associations between activity behaviours (physical activity, sedentary behaviour, and screen time) with mental well-being measures was also examined. The 10-item Kessler Psychological Distress Scale (K-10) (Kessler-10) was used as a global measure of psychological stress with scores 10–19 categorised as ‘Likely to be well’, 20–24 ‘Likely to have a mild mental disorder’, 25–29 ‘Likely to have a moderate mental disorder’ and 30–50 ‘Likely to have a severe mental disorder’ (30–50) [17]. The Kessler-10 is reported to be a valid and reliable population level instrument for psychological distress [17] and suitable within the Australian context [18, 19]. In the parent study [5] and this study’s subset, internal consistency (Cronbach’s alpha) for the Kessler-10 was α = 0.94 during lockdown and α = 0.94 post lockdown. Mental well-being was assessed using the 21-item Depression Anxiety Stress Scales (DASS-21) [20] as a measure of domains depression (7-items), anxiety (7-items) and stress (7-items) with a reported high internal consistency (depression α = 0.94, anxiety α = 0.87, stress α = 0.91) [21]. In the parent study [5] and this study’s subset, internal consistency for the DASS-21 was α = 0.97 during lockdown and α = 0.96 post lockdown. Scores were categorised from low to high severity and grouped for this study as normal, mild – moderate, and severe - extremely severe [20]. Loneliness was measured using the validated UCLA three-item scale [22] which reports good internal consistency (α = 0.72), with the summed score was categorised as No loneliness’ (3–4), ‘Moderate loneliness’ [5–7], and ‘Severe loneliness’ (8–9) [23]. In the parent study [5] and this study’s subset, internal consistency for Loneliness was α = 0.85 during lockdown and α = 0.84 post lockdown. Participants were asked to describe in an open-ended question what may have affected (positively or negatively) their mental well-being during the lockdown period.

Health promotion campaign recall was examined in the overarching study [5]. Relevant to the current study we examined open ended responses to “What health promotion messages should have been provided to the community during COVID-19 lockdown that were missing at the time?” to assess the relevance or potential importance of physical activity and well-being health promotion messaging.

Postcode was converted to a Socio-Economic Indexes for Areas (SEIFA) and categorised according to the Index of Relative Socio-economic Advantage and Disadvantage (IRSAD) [24]. For this study they were grouped into most advantaged, moderate advantage, moderate disadvantage and most disadvantaged. Body mass index (BMI) was calculated from self-reported height and weight (reported during and after lockdown) (kg/m^2^) and categorised as healthy < 25 kg/m^2^, overweight 25 to < 30 kg/m^2^ and obese ≥ 30 kg/m^2^.

### Statistical analysis

A demographic comparison of this study’s sample with those excluded from the original sample [5] was examined for sex and locality by Chi square (χ^2^) and for age by Mann-Whitney U (U). Data analysis was conducted using IBM SPSS version 27 (IBM, Chicago, IL, USA) with 2-sided p-values reported. Alpha < 0.05 was considered statistically significant.

Sex, SEIFA, locality (metro /rural), physical activity levels (very active/active/moderately active/low active/not active), physical activity days [1–7], and sedentary behaviour (mostly sitting/mostly standing/mostly walking/mostly heavy labour or physically demanding work) were described using frequency (f) and percent (%), with differences between the lockdown and post lockdown periods examined using McNemar-Bowker test (χ^2^). Age (years), time physical activity (days), non-work-related screen time (hours/week), and BMI (kg/m^2^) was described using mean (M), standard deviation (SD), median (Md) and interquartile range (IQR 25th to 75th percentiles).

A Wilcoxon signed rank test (W) examined differences between the lockdown and post lockdown periods for the number of days participants reported being physical active, BMI and non-work-related screen time. Chi-square tests (χ^2^) were used to examine physical activity levels with BMI categories at lockdown and at post-lockdown; while McNemar-Bowker test (χ^2^) was used to examine lockdown between group differences for sedentary behaviour; Kessler-10; DASS21 domains Stress, Anxiety and Depression; and Loneliness categories.

Changes of behaviour (post lockdown score minus during COVID lockdown score) were categorised as ‘no change’, ‘higher during lockdown’ or ‘lower during lockdown’. Chi-square analysis examined differences between changes in physical activity with BMI and each well-being outcome with Fisher-Freeman-Halton Exact Test χ2 and 2-sided exact p-value reported. Multinomial logistic regression was used to examine the association of physical activity changes for each of the mental well-being outcome’s changes and BMI. Category ‘no change’ was set as the comparison group. Confounding variables for all models included sex, SEIFA, age, and BMI difference (except for BMI model). SEIFA was excluded from DASS21 stress and anxiety model analyses due to lack of statistical power to run the procedure. Exp(β) with 95% confidence intervals (CI) with parameter Wald χ^2^ tests are reported for outcome categories ‘higher during lockdown’ and ‘lower during lockdown’.

Generalised estimating equations (GEE) were used to examine continuous dependent variables (physical activity days, non-work screen time), with participants treated as repeated. A binary logistic GEE examined active (very active/active/moderately active) versus inactive (not very active/ not active at all). Lockdown period and confounding variables: SEIFA, BMI category, and sex were treated as fixed factors, with age treated as a fixed covariate. The binary logistic GEE models were extended to examine the association with each mental well-being measure (included as fixed effects factors). For all models an interaction term was explored with lockdown period. A final binary logistic GEE model examined the relative influences of all well-being measures together on active versus inactive physical activity levels. Tests of model effects (Wald χ^2^) and parameter estimates (β or Exp(β)) with 95% confidence intervals (CI) are reported.

## Results

No significant difference was detected between participants included for this study (at least one physical activity measure) to those excluded (without physical activity measures) for sex (male n = 114 24.7% female n = 347 75.3% χ2 = 0.69 p = .404) or metro versus rural (metro n = 401 86.6% rural n = 62 13.4% χ2 = 3.33 p = .07). Included participants however were significantly (U = 3.92 p < .001) older (M = 49.3 SD = 16.3 Md = 51.0 IQR 36.0–61.0) compared to excluded participants (M = 41.0 SD = 17.9 Md = 39.0 IQR = 23.0–58.0). Specifically, the 18–24 years age group had a smaller representation (8.9%), while the 65 + years’ age group had a larger representation (19.4%) compared to those excluded (28.9% and 10.8% respectively). Excluded participants did not report their highest qualification, employment status or household income, so comparisons were not possible.

In this study, more than two-thirds of respondents were representative of advantaged SEIFA: most advantaged 52.6% (n = 284), moderate advantage 16.3% (n = 88); with less than one-third moderate disadvantage 26.1% (n = 141) and most disadvantaged 5.0% (n = 27). On average respondents were overweight and self-reported an increase in their BMI from time of lockdown (M = 26.3 SD = 5.8 Md = 25.0 IQR 22.2–29.4) to the post lockdown period (M = 26.7 SD = 6.1 Md = 25.4 IQR 22.3–30.5) (U = 3.0 p = .003). There was a slight but insignificant shift of more individuals to an obese weight category (24.5% during lockdown to 26.7% post lockdown) (Supplementary Fig. 1).

**Fig. 1:**
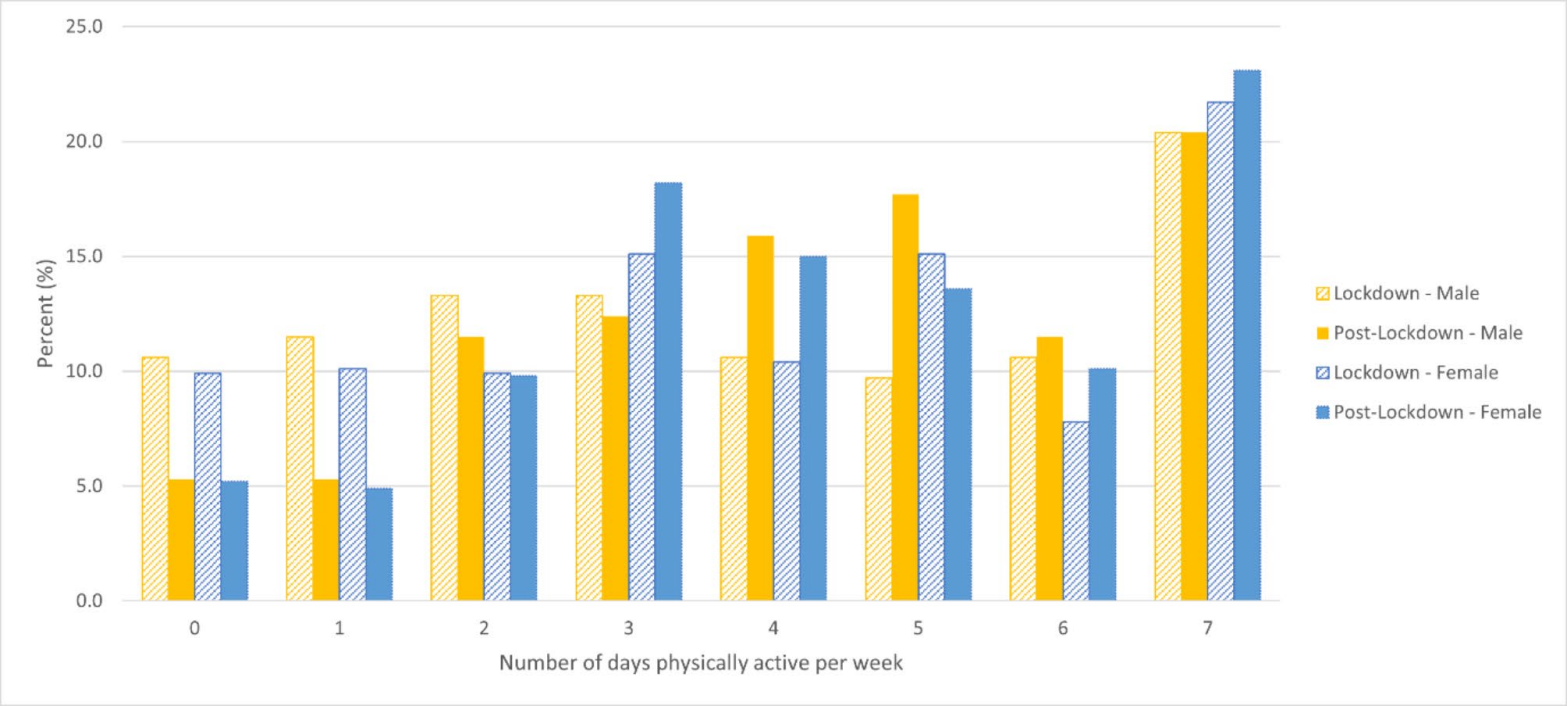
Trend of the number of days physically active during lockdown and post lockdown for total, males and females

**Fig. 2:**
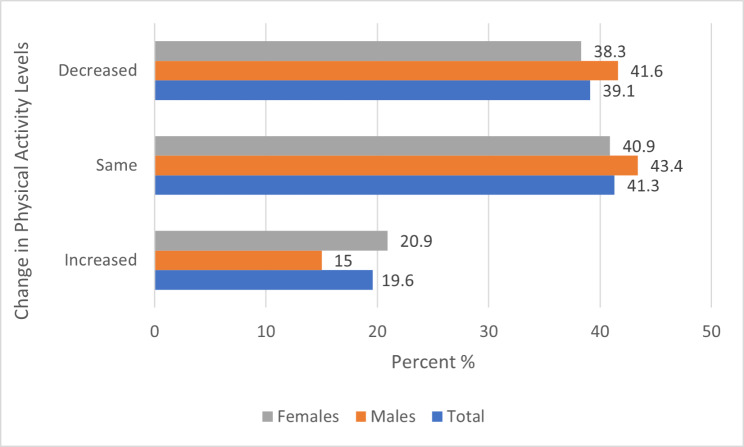
Change in physical activity days from lockdown to post lockdown for total, males and females

**Fig. 3:**
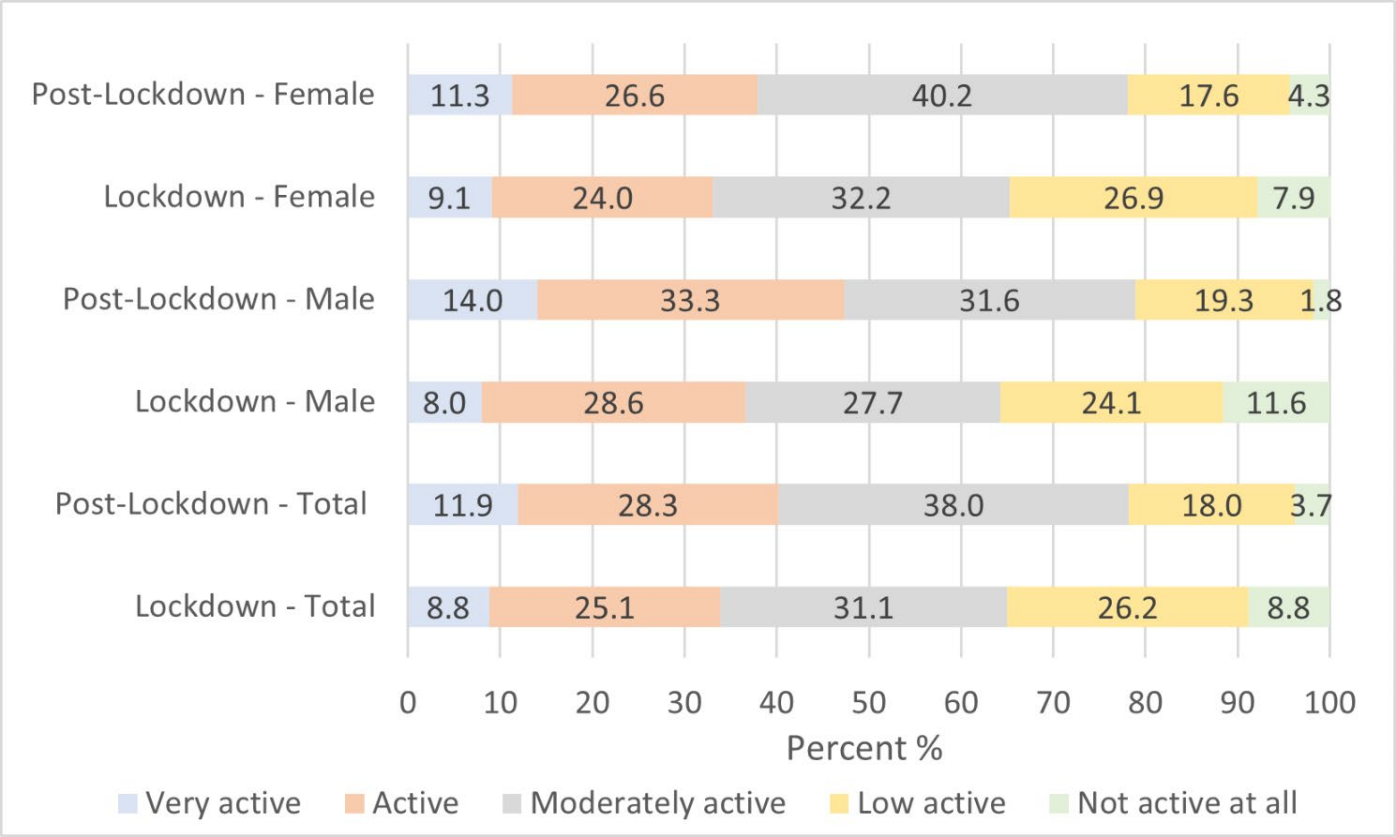
Summary of physical activity levels during lockdown and post lockdown for total, males and females

Participants spent significantly more days being physically active (W = 4.47 p < .001) post lockdown (M = 4.3 SD = 2.1 Md = 4.0 IQR 3.0–6.0) compared to during lockdown (M = 3.9 SD = 2.4 MD = 4.0 IQR 2.0–6.0). No differences between males and females were detected for number of days participants were physically active during lockdown (U = 0.65 p = .518) nor during post lockdown (U = 0.01 p = .994). Trends for the number of days physically active are depicted in Fig. 1. Emerging from lockdown, 41% of participants maintained their current physical activity levels, 39% lower levels and 20% higher levels (Fig. 2), with a summary of physical activity level categories: “very active” to “not active” depicted in Fig. 3.

GEE modelling for number of days of physical activity (Supplementary Table 1) confirmed lower activity levels during lockdown (Wald χ^2^= 13.6 p < .001), the obese group being the least active (Wald χ^2^= 10.1 p = .006), with higher activity levels with increasing age (Wald χ^2^= 27.3 p < .001). No statistically significant effect was detected for sex (Wald χ^2^= 0.3 p = .576) or SEIFA (Wald χ^2^= 0.6 p = .887). When activity levels were considered (active versus inactive), binary logistic GEE reported only significant effects for lockdown period (Wald χ^2^= 25.7 p < .001) and BMI categories (Wald χ^2^= 16.2 p < .001). Healthy weight and overweight individuals were both 2.8 times more likely to be active (Exp(β) CI 1.6–4.8 p < .001 and Exp(β) CI 1.5-5.0 p = .001 respectively) compared to obese individuals. During lockdown individuals were 2.3 times more likely to be inactive (Exp(β) CI 1.7–3.2 p < .001) compared to the post-lockdown period.

Specific between group differences were examined using multinomial logistic regression models controlling for confounders and are reported in Table 1. For sedentary screen time, DASS21 anxiety and DASS21 depression a significant inverse association was found. During lockdown, individuals who reported lower physical activity levels were 3.2 times more likely to report higher screen time (p < .001), 2.6 times more likely to report higher symptoms/indictors of anxiety (p = .027) and 2.5 times more likely to reported higher symptoms/indicators of depression (p = .011). Participants reporting higher physical activity levels during lockdown were 3.4 times more likely to report lower screen time (p = .015), 4.2 times more likely to report lower anxiety (p = .017) and 2.7 times more likely to report lower depression (p = .045). For Kessler-10, there was 2.2 risk of higher scores during lockdown, when physical activity levels were lower (p = .011). When controlling for confounders, no significant risks were detected for DASS21 stress or loneliness, although similar trends in risk were noted.

**Table 1:** The association of change in physical activity behaviours with change in well-being outcome measures from during lockdown to post lockdown

			Exp(β) 95% Wald Confidence Intervals			
Outcome Model^a^	Physical Activity^a^	Odds Ratio	Lower	upper	Wald χ2	p-value
**BMI**						
BMI higher in lockdown	Higher in lockdown	2.3	1.0	5.3	3.94	0.047*
	Lower in lockdown	2.1	1.0	4.6	3.62	0.057
BMI lower in lockdown	Higher in lockdown	1.2	0.5	2.6	0.12	0.730
	Lower in lockdown	3.4	1.8	6.7	13.17	< 0.001*
**Sedentary Screen Time**						
Screen Time higher in lockdown	Higher in lockdown	1.8	1.0	3.4	3.41	0.065
	Lower in lockdown	3.2	1.9	5.4	19.07	< 0.001*
Screen Time lower in lockdown	Higher in lockdown	3.4	1.3	8.9	5.90	0.015*
	Lower in lockdown	0.9	0.3	2.9	0.03	0.871
**Kessler-10**						
Kessler-10 higher in lockdown	Higher in lockdown	1.6	0.8	3.5	1.53	0.216
	Lower in lockdown	2.2	1.2	4.2	6.43	0.011*
Kessler-10 lower in lockdown	Higher in lockdown	1.6	0.5	4.7	0.59	0.443
	Lower in lockdown	0.8	0.3	2.4	0.19	0.663
**DASS-21 - Stress** ^**b**^						
Stress higher in lockdown	Higher in lockdown	0.9	0.3	2.7	0.01	0.922
	Lower in lockdown	1.8	0.8	4.0	2.12	0.145
Stress lower in lockdown	Higher in lockdown	1.2	0.4	3.6	0.16	0.692
	Lower in lockdown	0.4	0.1	1.3	2.50	0.114
**DASS-21 - Anxiety** ^**b**^						
Anxiety higher in lockdown	Higher in lockdown	1.3	0.4	4.1	0.18	0.672
	Lower in lockdown	2.6	1.1	6.2	4.90	0.027*
Anxiety lower in lockdown	Higher in lockdown	4.2	1.3	13.6	5.75	0.017*
	Lower in lockdown	1.1	0.3	3.7	0.01	0.915
**DASS-21 - Depression** ^**b**^						
Depressive symptoms higher in lockdown	Higher in lockdown	1.0	0.4	2.8	0.00	0.975
	Lower in lockdown	2.5	1.2	5.1	6.40	0.011*
Depressive symptoms lower in lockdown	Higher in lockdown	2.7	1.0	6.9	4.02	0.045*
	Lower in lockdown	0.6	0.2	1.9	0.62	0.431
**Loneliness**						
Loneliness higher in lockdown	Higher in lockdown	1.5	0.7	3.2	1.26	0.262
	Lower in lockdown	1.5	0.8	2.7	1.75	0.186
Loneliness lower in lockdown	Higher in lockdown	2.5	0.9	6.8	3.12	0.077
	Lower in lockdown	0.4	0.1	1.4	2.19	0.139

Note. PA = physical activity BMI = body mass index. ^1^ Multinomial Logistic Regression model controlling for sex, SEIFA, age and BMI difference (except BMI model which does not include BMI difference); ^b^ SEIFA excluded as a confounder from DASS-21 Stress and Anxiety models

Responses to open-ended questions regarding any changes in their physical activity during lockdown (Supplementary Table 2), indicated some respondents (percentage from analysis of results/themes) sought new ways to stay active (e.g., online classes), particularly as all team sports and group fitness were cancelled. However, many reported that the intensity of the training was reduced from vigorous or strength exercise to walking, running, yoga, or gardening. Some reported being ‘challenged’ to stay motivated and to find new ways to exercise in general or lacked motivation overall. Some gyms and fitness studios were reported by respondents to adapt to the lockdown by supporting their customers to stay fit in a number of ways. For some respondents, memberships were put on hold, cancelled, or some let memberships lapse. Some gyms offered free online courses and equipment hire to support members to stay active. There were also differences in how well people adapted to the change with some participants reporting they found it extremely difficult to return to normal, whereas some were able to pick up new ways to exercise that they continued practicing after lockdown.

Screen time open-ended question responses found a majority reported higher screen time during lockdown, although responses were more positive than negative. Technology was mainly used to reach out to family and friends. Overall, it seemed that people used the technology as a tool (e.g., entertainment, distraction, information). People signed up for different online courses, exercise, attended many online events (e.g., book clubs, church) in order to stay connected. A variety of leisure time activities were also reported, such as reading more books, doing puzzles, jigsaws or other crafts, baking, spending more time in the garden, or just doing extra work and projects around the house.

Overall, open ended responses indicated the change was ‘holistic’ and reasons for lower physical activity levels was multifaceted (Supplementary Table 2). Many also mentioned that they missed the social aspect of the exercise that had helped them to stay motivated before. However, many also tried their best to stay active during lockdown in order to maintain and improve their mental health. Some mentioned that this experience made them become more aware and to appreciate the little everyday things more.

Lastly, participants were also asked what type of health campaign they would like to see, under similar pandemic related lockdown. Many reported being relatively satisfied with the ongoing campaigns, while some required more detailed information and instructions regarding hygiene (e.g., mask wearing), virus and rationale behind the decision making, there was also a strong call for ‘*how to stay healthy during lockdown*’. Furthermore, many participants mentioned the need of mental health campaigns, covering all the aspects of mental well-being. Comments ranged across topics including:

- “*stress reduction techniques“ (Male, 38-yo, healthy weight)*,
- *“exercise (bring back Norm - Life be in it. )” (Female, 53-yo, healthy weight)*,
- *“Staying healthy at home etc.” (Male, 63-yo, healthy weight)*,
- *“good foods to eat when you’re not exercising much” (Female, 21-yo, healthy weight)*,
- *“mindful activities at home” (Female, 53-yo healthy weight)*,
- *“Specific strength training exercises that could be done in the home. Something like an app to encourage physical activity tracking – giving daily exercise suggestions and challenges and gamified to reward participation.” (Female, 52-yo, overweight*).

Some also highlighted targeting health promotion messaging to specific vulnerable groups such as people with disabilities, the elderly and those living alone.

Significantly higher non-work-related screen hours per week were reported during lockdown (M = 21.7 SD = 41.0 MD = 12.0 IQR 6.0–28.0) compared to post lockdown (M = 15.8 SD = 24.4 Md = 10.0 IQR 4.0–20.0) (W = 11.8 p < .001). No gender differences were detected for screen time during lockdown (U = 0.9 p = .353) nor during post lockdown (U = 1.2 p = .218). GEE modelling for non-work-related screen time (hours per week) (Supplementary Table 1) confirmed lower levels of screen time: post lockdown (Wald χ^2^= 6.0 p = .014), with the obese group (compared to healthy BMI) reporting the highest hours per week (Wald χ2 = 8.9 p = .012, Supplementary Fig. 1); and lower levels of screen time with increasing age (Wald χ^2^= 4.8 p = .028). No statistically significant effect was detected for sex (Wald χ^2^= 0.9 p = .338) or SEIFA (Wald χ^2^= 3.1 p = .374). A significant difference between sedentary behaviours was reported during lockdown and post-lockdown (χ^2^=28.4 p < .001), with higher levels of sitting time reported during lockdown. For females during lockdown, sitting time was replaced by more standing and walking post lockdown, while for men it was replaced with more time standing and heavy labour or physically demanding work (Supplementary Fig. 2).

Mental well-being results for the study sub-sample (Supplementary Table 3) were consistent with previously reported results on the full sample [5]. Overall post-lockdown most individuals reported being well (72.0% p < .001), had lower levels of mild-severe depression (27.7% p = .021), and were not lonely (45.2% p < .001) compared to during lockdown (60.5%, 35.0% and 30.8% respectively). No significant differences were detected for stress or anxiety.

## Discussion

This study provides an important contextual understanding of the physical and mental well-being associations the COVID-19 lockdown had on Western Australians thereby extending previous work [5]. Importantly, our results demonstrated a significant association between physical activity and mental well-being among Western Australians during COVID-19-lockdown. Our study’s mixed methodology approach provided further depth by exploring some of the drivers to these findings, demonstrating that there are multifaceted factors that each contribute to individual’s physical and mental well-being. These findings provide unique insights into successful strategies some individuals used to combat barriers to being physically active and managing well-being during COVID-19 lockdown.

Findings from this study indicated that overall physical activity levels were lower during lockdown. However, for one-fifth of individuals the lockdown period saw higher levels of physical activity. Although we used a different study design, our results align with lower physical activity levels being reported during a lockdown. Stanton et al. [9] reported that almost half of their respondents reported a reduction in physical activity since the onset of the COVID-19 pandemic, but about 20% also reported a positive change. Probable reasons for lower levels of physical activity during the lockdown include the closure of usual exercise venues, social distancing, the cessation of club and community sport and an unwillingness to change previous exercise habits; all of which have been offered previously by studies [9, 25].

Interestingly, our findings in regards to overall levels of physical activity being lower as a result of lockdowns differs from research which examined COVID-19 impacts with over 500 participants from 18 countries [26]. Brand et al. [26] found that many people maintained (44%), or increased (32%) and only 24% reported a decrease in exercise frequency. Whilst difficult to give exact details of each country’s COVID-19 restrictions in the above study, a possible explanation for this difference is that a number of the overall participants from the study were under restrictions for a longer period of time, sometimes for months at a time and in some cases for citizens of Italy and the UK, this occurred more than once. Potentially, people in countries affected by longer lockdown periods or rolling lockdowns were able to adapt to the restrictions. Kau et al. [27] lends support for this argument finding that during the initial phase of lockdown, participants reported a negative situational perception and a lack of motivation for fitness. However, over time there was a gradual improvement in positive self-perception and motivation to overcome their dependence on gym and fitness equipment which led to them continuing to exercise at home [27]. Furthermore, a government report produced in Australia reported a pattern emerged through COVID-19 where adults became more active on purpose with each wave of the pandemic [28].

An important finding in this study was a negative association between those who reported lower physical activity levels during lockdown and well-being measures; specifically, those who were less physically active during lockdown also reported significantly poorer levels of well-being. This finding is not surprising given Malcolm, Evans-Lacko [29] well-established link between physical activity and psychological well-being. Recent evidence in the form of an extensive meta-analysis (49 studies; N = 266,939) reported that physical activity can confer protection against the emergence of depression, regardless of age and geographical region [30], which our study findings support. In addition, Schuch, Vancampfort [30] stated that higher levels of physical activity are consistently associated with a lower odds of developing future depression and there is a need to emphasize the importance of policies targeting increased physical activity levels. Given the acknowledgement amongst Australian government agencies of the importance of mental well-being [31, 32], and the importance of physical activity to promote positive well-being, finding ways to overcome physical inactivity during pandemic-like situations is highlighted as a priority.

Qualitative findings from our study called for messaging around maintaining health during lockdowns. The challenge for those managing future lockdowns in response to pandemics or similar crisis is for government and health promoting agencies to ensure that there is messaging around the importance of physical activity for maintaining both physical and mental health. It appears that the people who would benefit most from this kind of messaging would be those who decreased physical activity levels during lockdown or who are inactive generally, regardless of whether there is a lockdown or not. One way of doing this, is to include educational material with specific information about the type of exercises people can do at home or in their backyard or garden to keep themselves physically active which in turn can then improve their mental health. The World Health Organisation (WHO) [2] state in their advice for the public regarding the pandemic, that it recognises it is hard to maintain the sort of exercise we normally do. They do include some recommendations about the amount of exercise. Also included in these recommendations are muscle-strengthening activities as part of daily physical activity on at least two days each week which can include push-ups, pull-ups, squats or lunges, lifting weights and household tasks that involve lifting, carrying or digging [3].

Whilst these recommendations are available, our study findings indicate more information through public messaging campaigns is needed particularly for those populations that are physically inactive (regardless of lockdown or not), and also for those whose physical activity levels decreased during the lockdown periods. Given the ongoing COVID-19 pandemic, this is important given decreased duration or frequency of physical activity may be associated with increased risk of mortality and hospitalisation for individuals infected with COVID-19 [33]. The information needs to be specific regarding types of exercises and activities that people could do, including videos that give example workouts and also demonstrate the correct technique; our open-ended responses demonstrate that this information was requested by some participants. Taking advantage of the higher screen times reported during lockdowns, these could then be uploaded to various websites and social media platforms to ensure the wider population has access, or even utilise the check-in style apps used during the COVID-19 pandemic. Furthermore, if this educational information comes from a local government or organisation that already has existing health promotion programs in place that includes context specific information, engagement could be further increased. An example of how this could work can be explored through using the Act-Belong-Commit (ABC) mental health promotion campaign in Western Australia, which is an evidence-based mental health promotion program that has been running since 2008 [34].

ABC targets individuals with respect to engaging in activities that strengthen and maintain good mental health; has a mass and targeted media presence; and is implemented through partnerships with local government, schools, workplaces, health services, state government departments, NGOs, hobby groups, local community organisations and sporting and recreational clubs [34]. For example, previously we stated that the Australian Government physical activity guidelines recommend strength related activities including push-ups, pull-ups, squats or lunges, lifting weights and household tasks that involve lifting, carrying or digging. For the population who regularly partake in exercise they may well know what these are; however, for those who are not regularly exercising or who may struggle to adapt from their gym routine to a home routine, more information than simply naming the exercise is needed. The creation of videos at a local level that are culturally appropriate and educate people about what these exercises are, how to correctly do them, suggestions for how you can create some weights at home (e.g., water filled milk cartons), which are then distributed through existing networks is well worth consideration.

Ding, Del Pozo Cruz [35] examined Google Trends throughout lockdown in Australia, the US, and the UK, and found that despite the challenges to an active lifestyle, there was an observed surge in community interest in exercise programs. This observed community interest needs to be transformed into people actively undertaking exercise, especially for those who are inactive or whose levels dropped. Our recommendation is that local government or health promoting agencies, who have a track record in delivering educational programs, develop appropriate resources to capture that interest and educate people about ways to be physically active. By providing avenues for a greater percentage of the population to engage in physical activity during lockdowns, the more people are receiving the protective factors that exercising provides which can be of benefit to both their physical and mental health.

The results of this study need to be considered in terms of the limitations of the retrospective design used, which relied on self-reported recall, as well as self-selection recruitment bias. Additionally, causation cannot be inferred without pre lockdown period data; the post lockdown period cannot be considered a proxy for pre lockdown as ongoing restrictions, albeit relaxed, continued. Despite exceeding the *a priori* sample size for the study, statistical power was likely a factor for examining the impact of mental well-being factors as the odds ratio suggested clinically important impacts that did not reach statistical significance. Further, the study was not powered to explore interaction effects, with results also suggesting that this might be important for some factors. There were people who reported minimum or no change in their physical activity due to the lockdown. However, physical activity levels before lockdown were not assessed as this was considered an excessive burden and would further improve recall bias.

## Conclusion

This study provides an important contextual understanding to the physical and mental well-being toll the COVID-19 lockdown had on Western Australians. Importantly, our results demonstrated a significant association between physical inactivity and mental well-being among Western Australians during and after the initial three-month COVID-19-Lockdown in March 2020. Our study’s mixed methodology approach highlighted there are multifaceted factors that each contribute to individual’s physical and mental well-being. These findings also provide recommendations into effective strategies future public health promotion campaigns could implement to combat barriers to being physically active.

## Electronic supplementary material

Below is the link to the electronic supplementary material.

Supplementary Material 1

Supplementary Material 2

## Data Availability

All data analysed during this study are available from the corresponding author on request and subject to appropriate HREC approvals.

## Abbreviations

HREC: Human Resource Ethics Committee
WA-HWSS: Western Australia Health and Well-being Surveillance system
DASS-21: Depression Anxiety Stress Scales
Kessler-10: Kessler Psychological Distress Scale (K-10)
