# Supplementary material for: Life in a time of COVID: retrospective examination of the association between physical activity and mental well-being in western Australians during and after lockdown
Source: BMC Public Health. 2023 Apr 14;23:701. doi: 10.1186/s12889-023-15440-1 (PMC10103040; doi:10.1186/s12889-023-15440-1)

Supplementary Table 1. Lockdown to post lockdown generalised estimating model estimates examining physical activity levels, number of days active, sedentary behaviour and screen time.

Model	Variable	β estimate	Standard Error	β 95% Wald Confidence Intervals		Wald χ2	p-value
				Lower	upper		
**Physical activity**	Intercept	1.83	0.47	0.91	2.75	15.1	<.001
(days per week)	Lockdown[a]	-0.47	0.13	-0.72	-0.22	13.6	***<.001**
	BMI: Healthy weight[b]	0.81	0.28	0.26	1.35	8.4	***.004**
	BMI: Overweight[b]	0.83	0.31	0.22	1.44	7.1	***.008**
	Sex: male[c]	0.15	0.27	-0.38	0.68	0.3	.576
	SEIFA: Most disadvantaged[d]	-0.01	0.62	-1.21	1.21	0.0	.982
	SEIFA: Moderate disadvantage[d]	-0.22	0.28	-0.78	0.34	0.6	.439
	SEIFA: Moderate advantage[d]	-0.01	0.33	-0.66	0.63	0.0	.969
	Age (years)	0.04	0.01	0.02	0.05	27.3	***<.001**
**Non-work Screen time**	Intercept	29.89	4.66	20.76	39.02	41.2	<.001
(hours per week)	Lockdown[a]	3.46	1.42	0.69	6.24	6.0	***.014**
	BMI: Healthy weight[b]	-8.90	3.04	-14.86	-2.94	8.6	***.003**
	BMI: Overweight[b]	-4.04	4.27	-12.41	4.33	0.9	.344
	Sex: male[c]	3.16	3.29	-3.29	9.61	0.9	.338
	SEIFA: Most disadvantaged[d]	3.32	5.06	-6.60	13.24	0.4	.512
	SEIFA: Moderate disadvantage[d]	-2.74	2.53	-7.70	2.23	1.2	.280
	SEIFA: Moderate advantage[d]	2.86	4.17	-5.30	11.03	0.5	.492
	Age (years)	-0.19	0.09	-0.35	-0.02	4.8	***.028**

Note. Comparison group [a]post lockdown, [b]obese, [c]female, [d]most advantaged,
* **bolded** indicate statistically significant effects p<.05.

Supplementary Table 2. Refer to separate word document.

Supplementary Table 3. Descriptiveness for mental well-being: psychological distress (Kessler-10), depression, anxiety and stress (DASS-21 domains) and loneliness (UCLA-3) during lockdown and post lockdown.

		Lockdown		Post lockdown		Lockdown difference	
**Mental Well-being tool**		**f**	**%**	**f**	**%**	**χ^2**	**P value**
**Kessler-10**						**37.6**	***<.001**
	Likely to be well (<20)	208	60.5	254	72.0		
	Mild mental disorder (20-24)	50	14.5	39	11.0		
	Moderate mental disorder (25-29)	36	10.5	22	6.2		
	Severe mental disorder (>30)	50	14.5	38	10.8		
**DASS 21 - Stress**						6.9	.077
	Normal	226	80.7	252	85.1		
	Mild - Moderate	39	13.9	24	8.1		
	Severe – Extremely severe	15	5.4	20	6.8		
**DASS 21 - Anxiety**						5.3	.151
	Normal	237	80.1	246	82.0		
	Mild - Moderate	30	10.1	28	9.3		
	Severe – Extremely severe	29	9.8	26	8.7		
**DASS 21 - Depression**						**9.7**	***.021**
	Normal	193	65.0	216	72.2		
	Mild - Moderate	68	22.9	56	18.7		
	Severe – Extremely severe	36	12.1	27	9.0		
**Loneliness**						**35.7**	***<.001**
	No loneliness	91	30.8	135	45.2		
	Moderate loneliness	90	30.5	77	25.8		
	Severe loneliness	114	38.5	87	29.1		

Note. * **bolded** indicates statistical significance p<.05. McNemar-Bowker test χ^2 reported.

Supplementary Figure 1. BMI boxplots for lockdown and post lockdown.

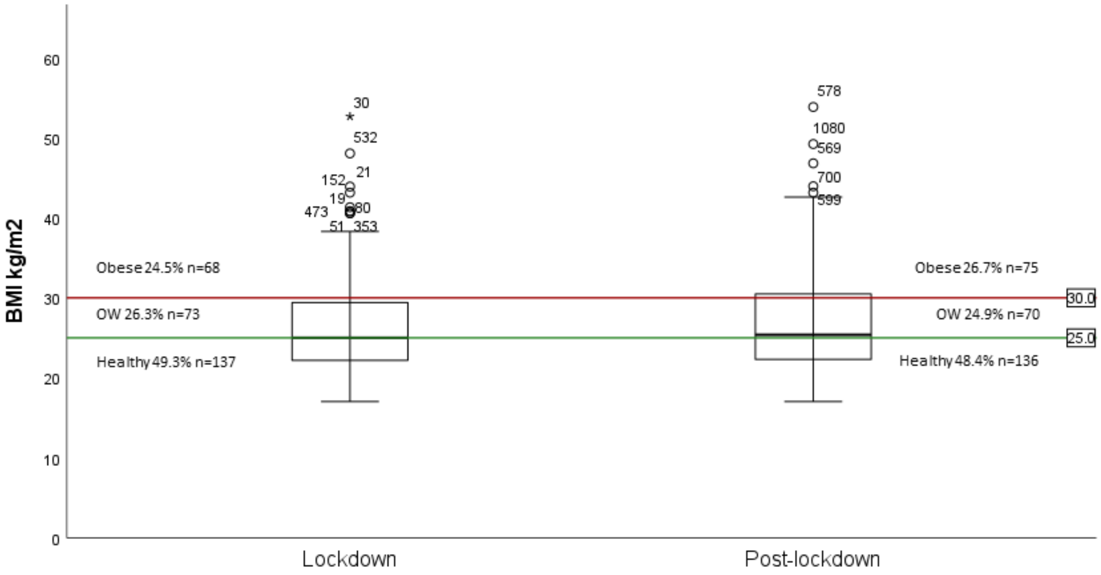

Note. BMI was calculated from self reported height and weight. Green line represents the cut-point between healthy and overweight (25 kg/m^2). Red line represents the cut-point between overweight and obese (30 kg/m^2). OW= overweight.

Supplementary Figure 2. Proportion of sedentary behaviours in lockdown and post lockdown for total, males and females.

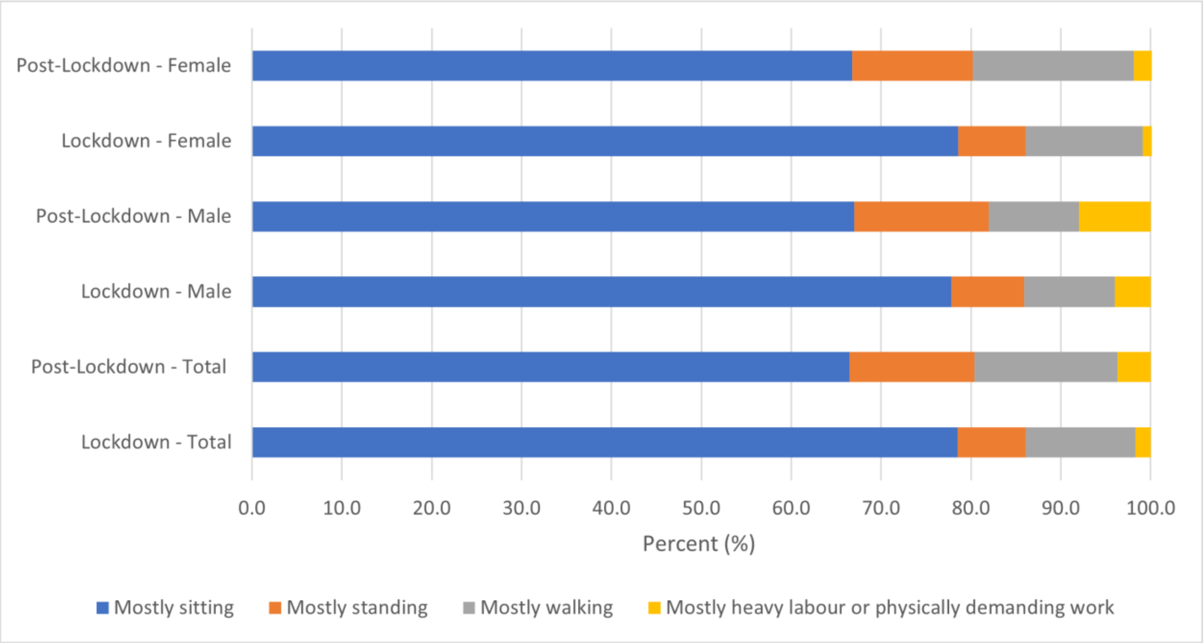

Supplement: Supplementary file 1 — Supplementary Material 1 [file 12889_2023_15440_MOESM1_ESM.pdf]
