# Supplementary material for: Life in a time of COVID: retrospective examination of the association between physical activity and mental well-being in western Australians during and after lockdown
Source: BMC Public Health. 2023 Apr 14;23:701. doi: 10.1186/s12889-023-15440-1 (PMC10103040; doi:10.1186/s12889-023-15440-1)
Supplement: Supplementary file 2 — Supplementary Material 2 [file 12889_2023_15440_MOESM2_ESM.pdf]

Table 2. Selection of open ended question responses to physical activity during lockdown and objective physical activity, screen time and well-being measures.

Quote	Weight change	Physical activity	Screen time	Psycho-logical	Stress	Anxiety	Depression
"Couldn't play competitive soccer or go to the gym so switched to running" (Male, 56-yo, overweight).	↑	↓	↑	↑	-	↑	↑
"I had to adapt to do my own workouts and moved to online workouts and programs which I quite enjoyed" (Female 27-yo, overweight)"	↓	-	-	-	-	-	-
"During COVID, I could not participate in team sports, so resorted to 'web' based yoga sessions instead. So my physical activity intensity during COVID changed to low intensity." (Female, 33-yo, overweight)	-	↓	↑	-	-	-	-
"My local pool was closed and so I could not attend my usual classes. I did not find any motivation to try an exercise at home or on my own." (Female, 60-yo, obese)	↑	↓	↑	↓	↓	↓	↓
"Due to the lockdown [sic] resulting my gym closed, I could not do my normal routing [sic], my physical activity level drooped [sic] dramatically. The change was partially the safety concern and lacking the motivation" (Female, 51-yo, healthy weight)	↑	↓	↑	-	-	-	-
"Couldn't go to gym Couldn't walk with friends Hate online classes" (Female, 40-yo, obese)	↑	↓	↑	↑	↑	↑	↑
"Club was closed, no motivation to do self workout" (Female, 36-yo, overweight)	↑	↓	↑	↑	↑	↑	↑
"I couldn't go to the gym which is where I usually get my physical activity from. Home workouts didn't work for me and at most I walked around the block." (Male, 21-yo, healthy weight)	↑	↓	↑	↑	-	-	↑
"Home workout program from my gym and gym gear was provided" (Male, 38-yo, healthy weight)	-	↓	-	-	-	-	-
"Gym fees on hold - on-line subscription provided free by Gym - overall cost saving" (Female, 59-yo, healthy weight)	-	-	↑	-	-	-	-

Quote	Weight change	Physical activity	Screen time	Psycho-logical	Stress	Anxiety	Depression
“Gym membership fees were reduced per month and moved to an online portal for members. Physical intensity I thought was similar to the gym, however I had to purchase dumbells for the workouts to increase the strength aspect of the training.” (Male, 48-yo, overweight)	-	-	-	-	↓	↓	↓
“I haven't picked back up the activity that I used to do pre lockdown, I don't go to the pool or go for power walks anymore.” (Female, 36-yo, overweight)	↑	↓	↑	↑		↑	↑
“I haven't started going back to the gym as yet. Not for any reason in particular, except that the habit has been broken and it's hard to get back in to it.” (OE_PA_3, #327, Female, 43-yo, overweight)	↑	-	↑	-	-	-	-
“I had never committed to exercise until February this year but now I enjoy pilates and walking, and I have participated in a weights/cardio workout through my employer. I always knew that it was important for mental health but I think this was especially true during lockdown in WA. So my exercise routine has stuck around." (Female, 35-yo, overweight)	-	↓	↑	-	-	-	↑
"I had to be more disciplined during lockdown as usually I do little exercise unless someone is there to push me. Given that, I tried a few different things and found that I liked the walking/running each day, which became a habit, and I've also started riding my bike on the weekends, which I love!" (Female, 60-yo, healthy weight, overweight)	↓	-	-	-	-	-	-
“Have had to make deliberate plans to walk everyday as I got out of the habit of 'moving' for exercise and enjoyment.” (Female, 44-yo, obese)	↑	↓	-	↑		↑	↑
“I did watch more TV but had it set up so I could watch while on the treadmill” (Female 27-yo, overweight)	↓	-	↑	↑	-	-	↑
“More time catching up with people. Learning to play the piano on an app” (Female, 49-yo, overweight)	-	-	↑	-	-	-	-
"Screen time increase as more leisure time available but less opportunity to participate; online offerings also went up so took part in new activities online e.g. cooking class, watching a theatre production; watched more Netflix; contact with family/friends didn't alter significantly apart from bookclub went online for one session; some volunteer activities such as meetings became online" (Female, 56-yo, normal weight/overweight)	↑	↓	↑	↑	-	-	↑
"I was reading a lot on social media and the news to stay connected with friends and the world around me, and I was also using my phone to call or FaceTime with friends, partly to socialise but partly to make sure everyone was coping okay. We did watch Netflix and Stan." (Female, 35-yo, overweight)	-	↓	↑	-	-	-	↑

Quote	Weight change	Physical activity	Screen time	Psycho-logical	Stress	Anxiety	Depression
“I was using screen time as a form of relaxation and killing time. I also used it to keep in touch with family and friends inter-state and overseas.” (Female, 52-yo, overweight)	↓	↑	↑	-	-	-	↑
Normally hate screens and only have it on in background. During lock down used it as distraction from stress. (Female, 40-yo, obese)	↑	↓	↑	↑	↑	↑	↑
“During lockdown, mostly watching news update regarding Covid condition and situation.” (OE_SED1, #237, Female, 51-yo, obese)	-	↓	↑	↑	-	-	-
“I watched less screen time purely because I was still working full time and studying online.” (Male, 28-yo, healthy weight)	↑	↑	-	-		↑	↓
No difference [leisure activity] – was still trying to maintain a routine (Male, 38yo, healthy weight)	-	↓	-	↑	↑	-	↑
No difference [leisure activity]. Any extra time I had I tried to spend reading, new hobbies and going outside. I had no extra screen time. This was not a trap that I wanted to fall into. (Female, 53-yo, healthy weight)	-	↑	↓	-	-	-	-
“During covid-19 my physical activity levels went completely rock bottom I noticed I wasn't eating the correct foods while still eating very little, no energy and my mental health took a swoop!" (Female, 21-yo, missing weight)	-	↓	↑	↑			
“closure of gyms resulted in a change of exercise. whilst trying to stay active at home it wasn’t the same. exercise changed from a highly active gym routine, to walking in a park. mental health also took a toll, which meant that exercise wasnt as interesting - so again decline” (Male, 38-yo, healthy weight)	-	↓	-	↑	↑	-	↑
"I think a big part of my motivation to exercise is the social element that I get from my gym, which I didn’t have during COVID-19. I also missed the one on one training with my PT (who has now left the industry due to the devastating effect COVID-19 had on her livelihood)." (Female, 58-yo, healthy weight)	↑	↓	↑	-	-	-	-
“It cancelled one of my groups, and eliminated face to face interaction in the other. These occurred as public health measures to prevent spread of COVID-19. Impact was little initially but over time it was negative, impacting my habits, physical & mental health, connectedness, motivation” (Male, 30-yo, healthy weight)	-	↓	↑	-	-	-	-
“I walked every day for mental health” (Female, 46-yo, healthy weight)	-	-	-	-	-	-	-

Quote	Weight change	Physical activity	Screen time	Psycho-logical	Stress	Anxiety	Depression
"Have had to make deliberate plans to walk everyday as I got out of the habit of 'moving' for exercise and enjoyment." (Female, 44-yo, obese)	↑	↓	-	↑	↑	↑	↑
"I decided to exercise more do ease my mentally relax and to improve health. The Covid-19 lockdown was mentally tiring due to constant attention to Covid-19-related news, a generally heightened alertness inside and outside in the community and the need for a new kind of routine." (Male, 30-yo, healthy weight)	↓	↑	↑	-	-	↓	↓
"It has made me try and make more time to exercise than I did before, go down the beach more and nature walks as it was enjoyable and good to destress and take time out from my usually hectic life" (Female, 51-yo, healthy weight)	-	-	↑	-	↓	-	↓
"I had never committed to exercise until February this year but now I enjoy pilates and walking, and I have participated in a weights/cardio workout through my employer. I always knew that it was important for mental health but I think this was especially true during lockdown in WA. So my exercise routine has stuck around." (Female, 35-yo, overweight)	-	↓	↓	-	-	-	↓
"I discovered I enjoyed walking regularly. Used this as a means for catching up with friends - fresh air, burning calories, wasn't spending money on cafe coffee. A win-win-win. I changed my way of thinking - from focussing too much time on work, to learning to take time out for myself, exercising more." (Female, 53-yo, overweight)	-	-	-	-	-	-	-

Note: ↑ higher; ↓ lower; – stable; blank indicates miss
